# Association between aquaporin 3 and transforming growth factor-beta 1 levels in decidual tissue and serum of patients with missed abortion

**DOI:** 10.3389/fmed.2025.1540257

**Published:** 2025-05-01

**Authors:** JinLing Yuan, JianXin Dong, Wanting Li, YingMo Zu, YanJuan Guo, Ying Zhang, Yan Chen

**Affiliations:** ^1^Gynaecology and Obstetrics,North China of Science and Technology University Affiliated Hospital, Tangshan Hebei, China; ^2^Guangzhou University of Chinese Medicine, Guangdong Guangzhou, China; ^3^Gynaecology and Obstetrics, The First Hospital of Qinhuangdao, Qinhuangdao Hebei, China; ^4^Beijing Obstetrics and Gynecology Hospital, Capital Medical University, Capital Medical University, Gynecology Minimally Invasive Center, Beijing, China

**Keywords:** missed abortion, induced abortion, decidual tissue, TGF-beta 1, AQP3

## Abstract

**Objective:**

To investigate the expression of aquaporin 3 (AQP3) and transforming growth factor-β1 (TGF-β1) in the decidual tissue and serum of patients with missed abortion (MA) and explore their clinical significance, evaluating their potential as diagnostic biomarkers for MA.

**Methods:**

A total of 40 MA patients (case group) and 40 induced abortion (IA) patients (control group) were included. Immunohistochemistry (IHC), Western blot (WB), and reverse transcription quantitative PCR (RT-qPCR) were used to detect the protein and mRNA expression of AQP3 and TGF-β1 in decidual tissue. Serum levels of AQP3 and TGF-β1 were measured by ELISA. The diagnostic efficacy was assessed using receiver operating characteristic (ROC) curve analysis.

**Results:**

The protein expression of AQP3 and TGF-β1 in the decidual tissue of the MA group was significantly higher than that of the IA group, with a 2.3-fold and 2.5-fold increase, respectively (*p* < 0.01), and their mRNA expression was also significantly upregulated (*p* < 0.01). Serum levels of AQP3 and TGF-β1 increased by 2.3-fold and 3.3-fold, respectively (*p* < 0.01). ROC analysis demonstrated that serum AQP3 (AUC = 0.887) and TGF-β1 (AUC = 0.949) exhibited high diagnostic accuracy for MA, with the combined detection achieving an AUC of 0.976, sensitivity of 92.5%, and specificity of 97.5%.

**Conclusion:**

AQP3 and TGF-β1 are significantly overexpressed in the decidual tissue and serum of MA patients and may play a role in the pathogenesis of MA by regulating trophoblast function. The combined detection of these two biomarkers holds promise as potential diagnostic tools for MA, offering new directions for early clinical management.

## 1 Introduction

Missed abortion (MA) is a type of spontaneous abortion characterized by the intrauterine death of an embryo or fetus before 20 weeks of gestation, without external intervention or the expulsion of the products of conception (1, 2). MA accounts for up to 15% of spontaneous miscarriages in clinically recognized pregnancies, and its incidence is rising (3, 4). Several epidemiological factors contribute to MA, including chromosomal abnormalities, immune disorders, hereditary thrombophilia, uterine abnormalities, infections, endocrine disorders, and environmental influences (5, 6). Of these, chromosomal abnormalities—such as numerical errors, copy number variations, and structural defects—are the most significant, accounting for 60–80% of cases (7, 8). The pathogenesis of MA remains complex, with placental dysfunction being a key contributor. Insufficient angiogenesis and excessive trophoblast apoptosis are central to the placental etiology of MA (9, 10). However, in 20–40% of cases, the underlying causes remain unknown, indicating that further research is needed to fully understand the mechanisms involved (5).

Placental development begins with the trophectoderm cells, which differentiate into cytotrophoblasts, syncytiotrophoblasts, and extravillous trophoblasts (EVTs) (11, 12). In early pregnancy, the proper invasion of EVTs into the uterine decidua, myometrium, and spiral arteries is essential for endovascular remodeling, ensuring adequate maternal-fetal blood circulation (13, 14). Abnormal EVT invasion has been linked to various pregnancy complications, such as fetal growth restriction, preeclampsia, recurrent early miscarriage, and MA (15–17). Despite these associations, the precise mechanisms by which EVT dysfunction leads to MA are not yet fully understood.

Aquaporin 3 (AQP3), a transmembrane channel protein in the aquaporin family, plays a critical role in water transport and cellular proliferation (18, 19). AQP3 is expressed in a variety of tissues, including the placenta and fetal membranes, and is involved in follicular development, blastocyst formation, embryo implantation, and fluid balance between the mother and fetus (20, 21). Abnormal AQP3 expression has been associated with pregnancy disorders such as gestational diabetes mellitus and preeclampsia (22, 23). Additionally, reduced AQP3 expression has been shown to impair EVT migration, suggesting that AQP3 is essential for normal trophoblast function (24). Given this, abnormal AQP3 expression may contribute to placental dysfunction and MA, though this relationship has not been thoroughly investigated.

Transforming growth factor-beta (TGF-β), a cytokine that regulates cellular homeostasis, growth, differentiation, and apoptosis, is also implicated in pregnancy outcomes (25, 26). TGF-β1, the first identified member of the TGF-β family, regulates trophoblast invasion and endometrial function (27, 28). *In vitro* studies have demonstrated that TGF-β1 induces apoptosis in decidual cells, affecting embryo implantation and leading to adverse pregnancy outcomes (29). Additionally, TGF-β1 has been shown to upregulate AQP3 expression via the EGF/EGFR pathway, which is associated with tissue hydration and wound healing (30, 31). Given these roles, it is possible that dysregulation of both AQP3 and TGF-β1 contributes to the pathogenesis of MA, although this has not yet been reported.

In this study, we investigated the expression of AQP3 and TGF-β1 in decidual tissue from cases of MA and induced abortion (IA) using immunohistochemistry (IHS), western blotting (WB), and reverse transcription-quantitative PCR (RT-qPCR). Serum levels of AQP3 and TGF-β1 were also measured using ELISA. By examining the association between these factors and MA, we aim to deepen our understanding of the pathophysiology of MA and identify potential biomarkers for early clinical diagnosis.

## 2 Materials and methods

### 2.1 Patients

This case–control study enrolled 40 women with missed abortion (MA, cases) and 40 women undergoing induced abortion (IA, controls), matched for age (±2 years) and gestational age (±1 week). The study was approved by the Ethics Committee of North China University of Science and Technology Affiliated Hospital (Ethics Certificate No. 20240313050). All participants were treated at the Department of Obstetrics and Gynecology of the hospital between October 2017 and May 2018. Written informed consent was obtained from all participants for the use of decidual tissue, serum samples, and clinical data.

#### 2.1.1 Group definitions and diagnostic criteria

1. MA Group Diagnostic Criteria (Missed Abortion, *n* = 40):

Intrauterine embryonic demise confirmed by transvaginal ultrasound at 6–12 weeks of gestation, meeting at least one of the following:

- Gestational sac mean diameter (MSD) ≥ 25 mm with no yolk sac or embryonic structures.
- Absence of fetal cardiac activity with crown-rump length (CRL) ≥ 7 mm.
- No growth of the gestational sac or appearance of fetal cardiac activity after ≥7 days of serial monitoring.

Exclusion of Other Etiologies: Cases were excluded if caused by ectopic pregnancy, infection, trauma, or coagulopathy (confirmed by medical history and laboratory tests).

2. IA Group Inclusion Criteria (Induced Abortion Control, *n* = 40):

- Voluntary termination of pregnancy without medical indications (e.g., fetal abnormalities or maternal health risks).
- Ultrasound-confirmed intrauterine viable pregnancy (normal fetal cardiac activity and regular gestational sac morphology).

#### 2.1.2 Common inclusion criteria (applied to both MA and IA groups)

1. Baseline Demographic Characteristics:

- Age 18–40 years, with MA and IA groups matched for age (±2 years), gestational age (±1 week), and BMI (±10%).
- Singleton pregnancy at 6–12 weeks of gestation (calculated by last menstrual period or ultrasound CRL).

2. Exclusion Criteria:

- Pregnancy Complications: Placental abnormalities (placenta previa, placenta accreta) or threatened abortion symptoms (vaginal bleeding ≥ menstrual flow).
- Infections and Chronic Diseases: Active genital tract infections (vaginitis, cervicitis), systemic infections (HIV, HBV, tuberculosis), endocrine disorders (diabetes, thyroid dysfunction), or autoimmune diseases (antiphospholipid syndrome, systemic lupus erythematosus).
- Medication and Behavioral Factors: Use of hormonal medications (contraceptives, glucocorticoids) within the past 3 months, smoking (≥5 cigarettes/day), or alcohol abuse (≥14 units/week).
- Recurrent Miscarriage History: ≥2 spontaneous abortions (exclusion applied only to the IA group; MA group was exempt).

### 2.2 Decidua tissue collection

Decidual tissues were obtained through artificial abortion using negative pressure suction and subsequently washed with normal saline. A portion of the samples was stored at −80°C for future Western blotting and RT-qPCR analyses, while the remaining samples were fixed in 10% formalin for immunohistochemistry analysis.

### 2.3 Serum collection

Within 1 h prior to the artificial abortion, 5 mL of peripheral venous blood was drawn from the cubital vein using a syringe and placed into a coagulation-promoting tube. The serum was then separated by centrifugation at 3,000 rpm for 10 min at 4°C, sealed, and stored at −80°C for subsequent analysis of AQP3 and TGF-β1 levels.

### 2.4 Biochemical analyses

Clinical examination data were collected from the Department of Obstetrics and Gynecology at North China University of Science and Technology Affiliated Hospital. Routine blood tests, including hemoglobin and white blood cell counts, were conducted by the hospital’s Laboratory Department. Serum levels of AQP3 and TGF-β1 were measured using commercial ELISA kits (catalog no. SP11203 for AQP3 and SP10208 for TGF-β1; Wuhan Saipei Biotech Co., Ltd.), following the manufacturer’s instructions.

### 2.5 Immunohistochemistry analysis

Decidual tissue samples fixed in 10% formalin were successively dehydrated, embedded in paraffin, and sectioned into 4 μm slices. After routine dewaxing and rehydration, antigen retrieval was performed in sodium citrate buffer (Solarbio, Beijing Solarbio Science & Technology Co., Ltd.). Sections were incubated overnight at 4°C with primary antibodies against AQP3 (catalog no. YLA1093HU, Shanghai YL Biotech Co., Ltd.; dilution 1:200) or TGF-β1 (catalog no. YLA0886HU, Shanghai YL Biotech Co., Ltd.; dilution 1:30). The following day, sections were incubated with goat anti-rabbit IgG horseradish peroxidase (HRP)-conjugated secondary antibody (catalog no. ZB-2301; ZSGB-BIO) at 37°C for 30 min. Staining was developed using a DAB kit (ZSGB-BIO), according to the manufacturer’s instructions. Negative controls were prepared by omitting the primary antibody.

Immunoreactive signals for both AQP3 and TGF-β1 were mainly located in the cytoplasm of decidual cells. Images of stained sections were captured at 400 × magnification using a Micro Publisher 5.0 microscope (Roper Industries). For quantitative image analysis, we imported the images into Image-Pro Plus 6.0 software (Media Cybernetics, Inc.). In three randomly chosen high-power fields per section, integrated optical density (IOD) of the positively stained area was measured after uniform background subtraction. The mean IOD from these fields was used as the final value for each sample. These quantitative data were then subjected to statistical analysis.

### 2.6 Western blotting

Total cytoplasmic protein was extracted from decidual tissue and quantified using a BCA Protein Assay Kit (Beyotime, Beyotime Biotechnology) according to the manufacturer’s instructions. Protein samples (20 μL per lane) were denatured and separated using SDS-PAGE (Zomanbio, Beijing Zoman Biotechnology Co., Ltd.), followed by transfer to a PVDF membrane at 30 mA for 12 h. To block non-specific binding sites, the PVDF membranes were incubated with 5% skimmed milk at 4°C overnight. The membranes were then probed with primary antibodies: rabbit anti-human AQP3 (dilution 1:10; catalog no. YLA1093HU; Shanghai YL Biotech Co., Ltd.), rabbit anti-human TGF-β1 (dilution 1:30; catalog no. YLA0886HU; Shanghai YL Biotech Co., Ltd.), and rabbit anti-human β-actin (dilution 1:10; catalog no. AF7018; Affinity BioReagents, Inc.) for 2 h at 37°C. Afterward, the membranes were incubated with goat anti-rabbit IgG HRP-conjugated secondary antibodies (dilution 1:2000; catalog no. ZB-2301; ZSGB-BIO, Beijing Zhongshan Goldenbridge Biotechnology Co., Ltd.) for an additional 2 h at 37°C. Protein signals were detected using an ECL Chemiluminescence Kit (Beyotime, Beyotime Biotechnology) and analyzed with Image Lab software (Bio-Rad Laboratories, Inc.). The AQP3 and TGF-β1 signals were normalized to the β-actin signal to obtain their relative expression levels.

### 2.7 RT-qPCR

Total RNA from decidual tissue was extracted using TRIzol reagent (Mei5bio; Beijing Jumei Biotechnology Co., Ltd.) and subsequently reverse transcribed into cDNA using the RevertAid First Strand cDNA Synthesis Kit (Mei5bio) following the manufacturer’s instructions. Quantitative PCR (qPCR) was performed using the SYBR Green Realtime PCR Mix kit (Mei5bio), with β-actin serving as the internal reference. The primers used for amplification were as follows: AQP3 forward: 5′-GAA GTC AGG TCA TAA GTT 3′ and reverse: 5′-CAT TGT TGA GTA GAG GAT-3′; TGF-*β*1 forward: 5′-CGT GCT AAT GGT GGA AAC-3′ and reverse: 5’-GCT CTG ATG TGT TGA AGA AC-3′; β-actin forward: 5′-ACT CTT CCA GCC TTC CTT-3′ and reverse: 5′-ATG TCC ACG TCA CAC TTC-3′. The PCR reactions were conducted in a total volume of 20 μL, with the following cycling conditions: 95°C for 15 s, 65°C for 15 s, and 72°C for 60 s, for a total of 40 cycles. The relative expression levels of AQP3 and TGF-*β*1 mRNA were calculated using the 2^−ΔΔCt^ method, where *Δ*Ct is defined as the Ct value of AQP3 or TGF-β1 minus the Ct value of β-actin, and *ΔΔ*Ct is the difference between the Ct values of MA and IA.

### 2.8 Statistical analysis

All statistical analyses were performed using SPSS 22.0 software, and graphical representations were created with GraphPad Prism 9 software. Normally distributed data were analyzed using independent samples t-test, while non-normally distributed data were analyzed using the Mann–Whitney U test (or Kruskal-Wallis test, depending on the situation). Diagnostic performance was assessed using receiver operating characteristic (ROC) curves to evaluate sensitivity, specificity, and their corresponding optimal cutoff values. Data are presented as mean ± standard deviation (mean ± SD), and differences between groups were considered statistically significant when the *p* value was less than 0.05.

## 3 Results

### 3.1 Clinical data analysis

Between MA and IA groups, there were no significant differences in age, BMI, hemoglobin, A history of upper respiratory tract infection past medical history, and spontaneous abortion (*p* > 0.05, Table 1).

**Table 1:** The clinical data analysis results of MA and IA groups (mean ± SD).

Characteristics	MA (*n* = 40)	IA (*n* = 40)	*P*
Age (years)	28.95 ± 4.48	30.00 ± 0.45	0.144
BMI	23.11 ± 3.26	22.20 ± 2.74	0.180
Hemoglobin (g/L)	135.40 ± 20.40	137. 40 ± 12.07	0.595
A history of upper respiratory tract infection	2	1	0.556
Past medical history	3	1	0.305
Spontaneous abortion	16	15	0.818

### 3.2 Expression of AQP3 and TGF-β1 in decidual tissue and serum, and their potential as biomarkers for MA

**Figure 1:**
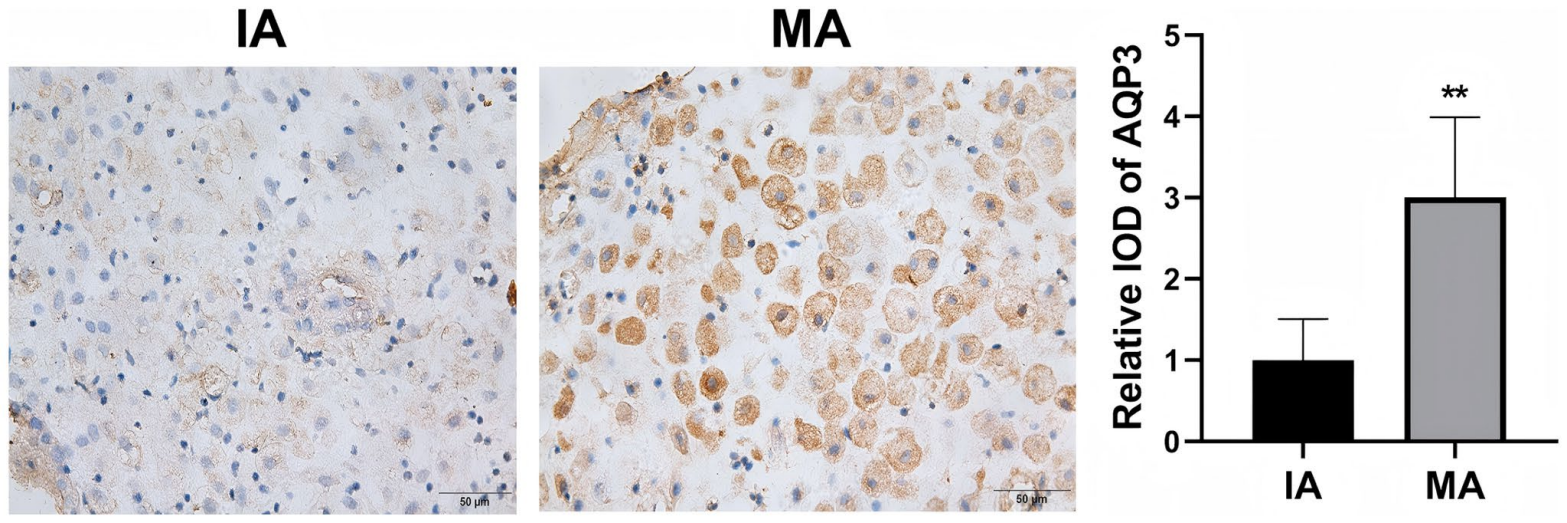
Immunohistochemical detection of AQP3 protein expression in decidual tissue. **p < 0.01 vs. IA. *n* = 40.

**Figure 2:**
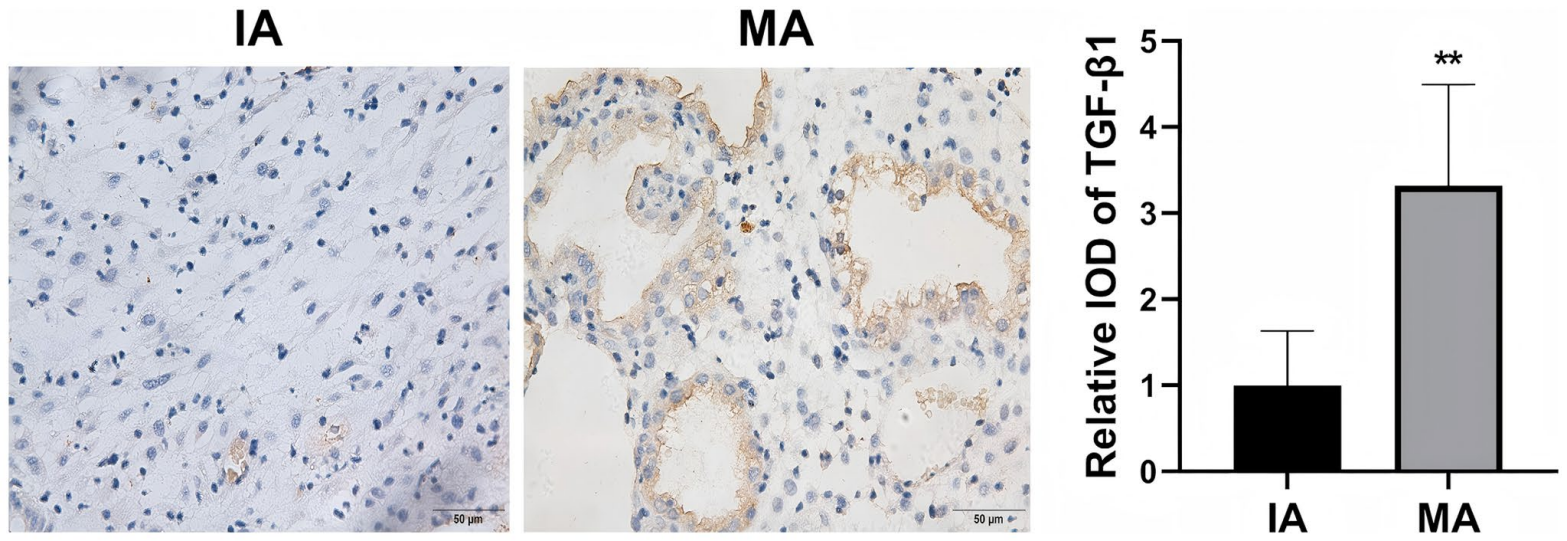
Immunohistochemical detection of TGF-β1 protein expression in decidual tissue. **p < 0.01 vs. IA. *n* = 40.

**Figure 3:**
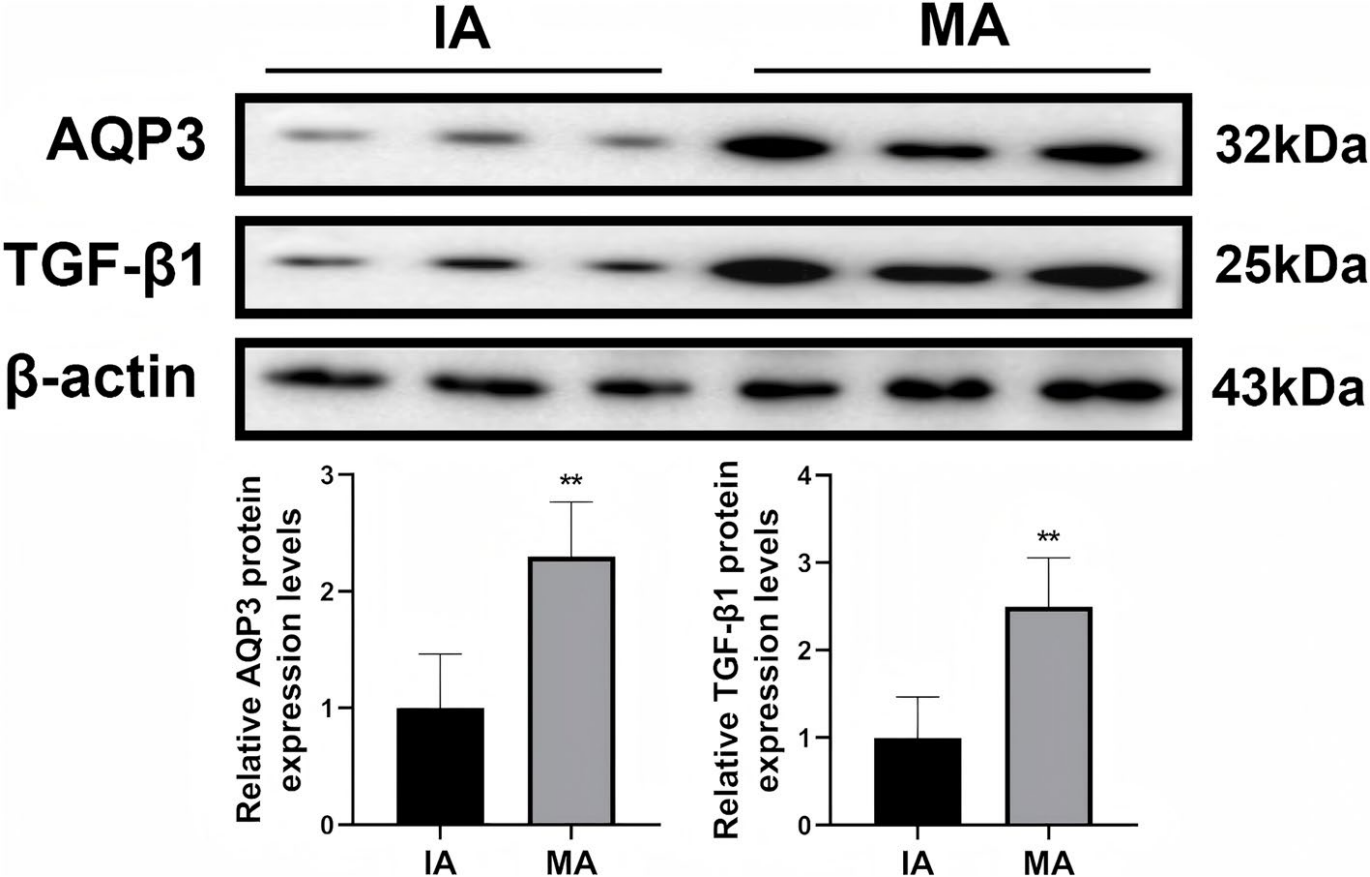
Western blot analysis of AQP3 and TGF-β1 protein expression in decidual tissue. Each lane corresponds to one randomly selected patient sample (3 patients from the IA group and 3 from the MA group). **p < 0.01 vs. IA. *n* = 40.

We first assessed the expression levels of AQP3 and TGF-β1 in decidual tissue through immunohistochemistry (IHC) (Figures 1, 2). AQP3 was predominantly localized in the cytoplasm of decidual stromal cells, showing widespread positivity across most cells in the tissue. In contrast, TGF-β1 was primarily observed in endothelial cells. The overall protein levels of both AQP3 and TGF-β1 were significantly higher in the MA group than in the IA group (*p* < 0.01). Western blot results were consistent with the IHC findings, demonstrating 2.3-fold and 2.5-fold increases in AQP3 and TGF-β1 expression, respectively, in the MA group compared to the IA group (*p* < 0.01, Figure 3).

**Figure 4:**
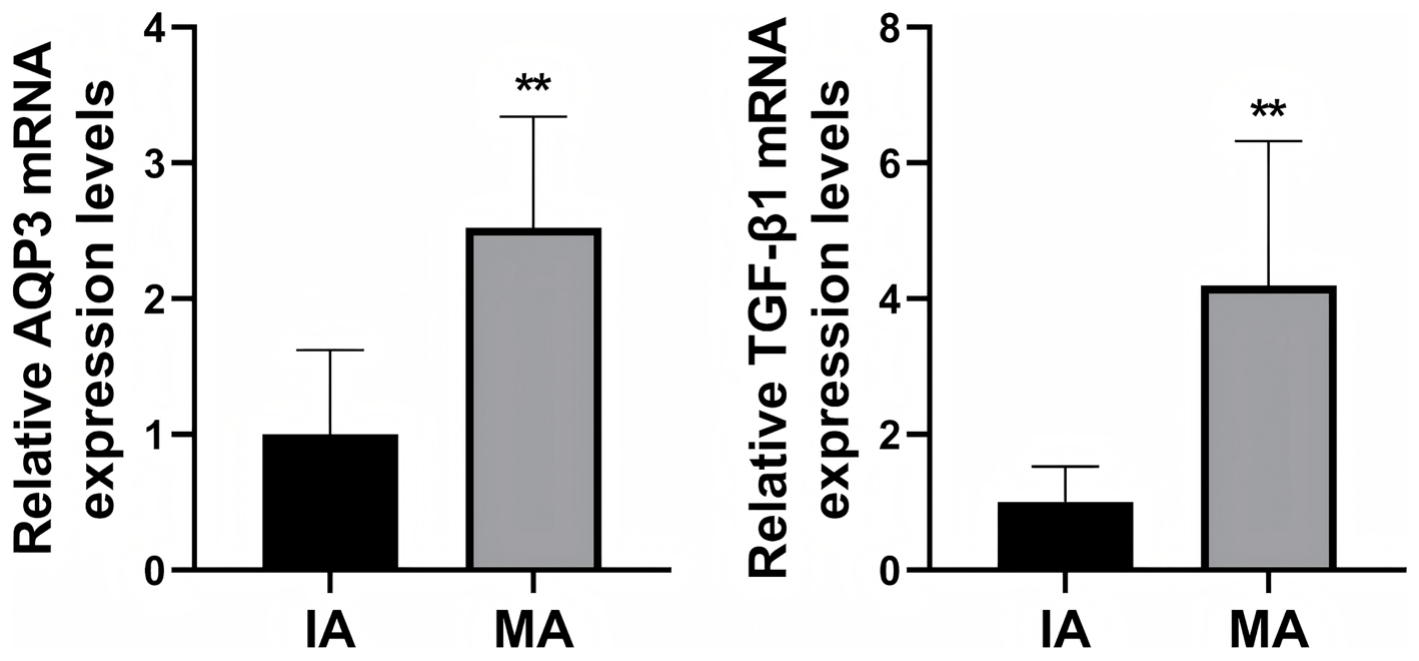
RT-qPCR analysis of AQP3 and TGF-β1 mRNA expression in decidual tissue. **p < 0.01 vs. IA. *n* = 40.

To further validate the role of AQP3 and TGF-β1 in MA, we performed RT-qPCR to measure the mRNA expression levels of AQP3 and TGF-β1 in decidual tissue from both groups (Figure 4). The results demonstrated that the relative mRNA expression levels of AQP3 and TGF-β1 were significantly higher in the MA group compared to the IA group (*p* < 0.01), further supporting the potential involvement of AQP3 and TGF-β1 in the pathogenesis of MA.

**Figure 5:**
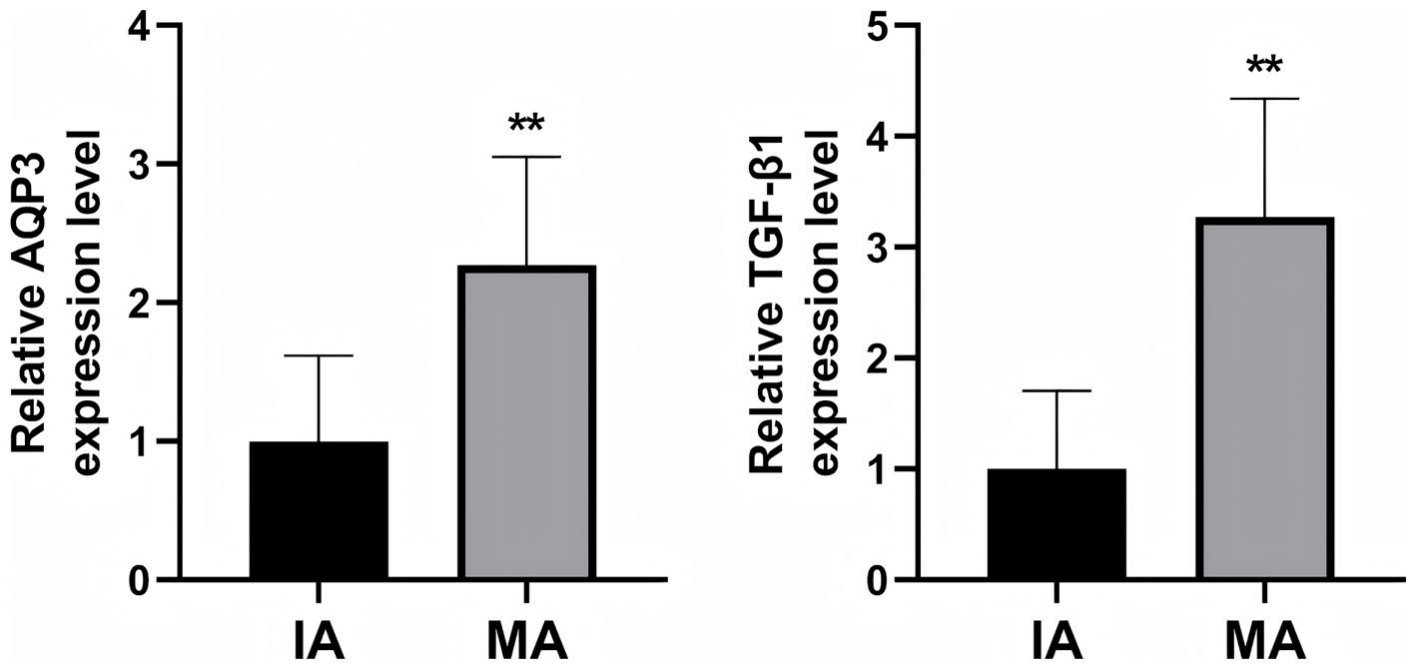
ELISA detection of AQP3 and TGF-β1 expression in patient serum. **p < 0.01 vs. IA. *n* = 40.

To explore whether AQP3 and TGF-β1 could serve as serum biomarkers for MA, we measured their serum levels using ELISA (Figure 5). The results indicated that the serum levels of AQP3 and TGF-β1 were significantly higher in the MA group, with increases of 2.3-fold and 3.3-fold, respectively, compared to the IA group (*p* < 0.01). These findings suggest that serum AQP3 and TGF-β1 may serve as potential biomarkers for the diagnosis of MA.

**Figure 6:**
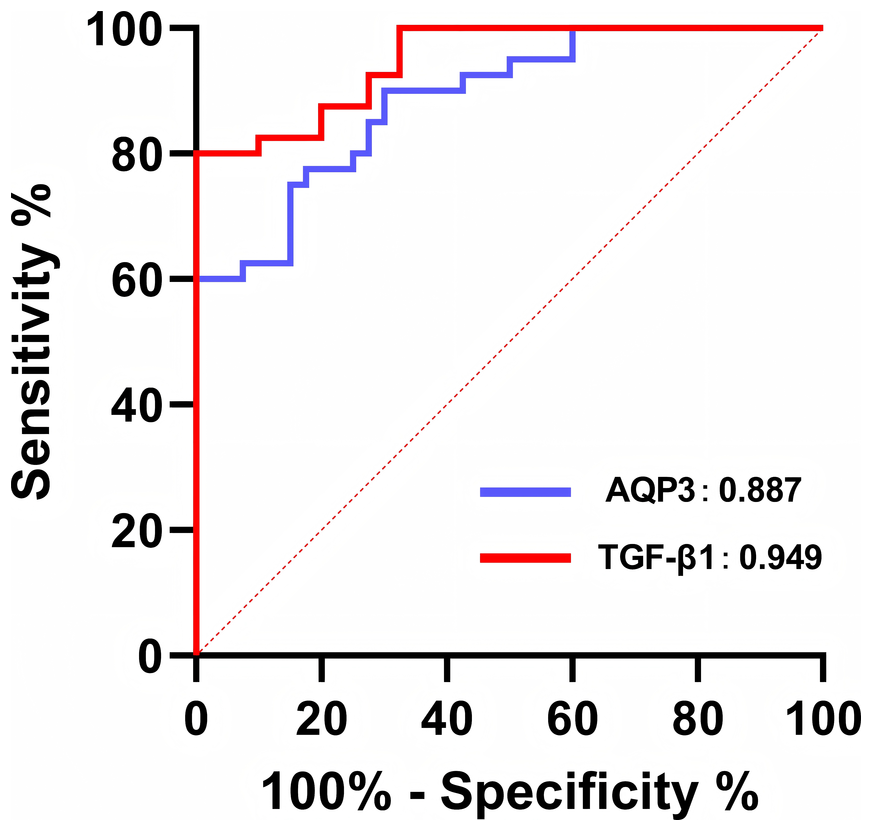
ROC curve of serum AQP3 and TGF-β1 for the diagnosis of MA.

To further test this hypothesis, we conducted ROC analysis to evaluate the diagnostic accuracy of serum AQP3 and TGF-β1 levels as risk predictors for MA (Figure 6; Table 2). The results showed that both AQP3 (AUC = 0.8869) and TGF-β1 (AUC = 0.9494) exhibited high diagnostic accuracy, indicating the potential of these biomarkers in predicting MA. However, the ROC analysis of AQP3 revealed multiple thresholds with the same maximal Youden index, making it difficult to select a single optimal threshold. Therefore, we opted to combine AQP3 and TGF-β1 as diagnostic markers to improve prediction accuracy.

**Table 2:** Diagnostic value of serum AQP3, TGF-β1, and the combined detection of AQP3 + TGF-β1 for MA.

Index	Cutoff value	AUC	*P* value	Sensitivity %	Specificity %
AQP3	1.75 ng/L	0.887	<0.01	90	70
	2.32 ng/L			77.5	82.5
	2.41 ng/L			75	85
	3.05 ng/L			60	100
TGF-β1	1.94 ng/L	0.949	<0.01	80	95
AQP3 + TGF-β1	0.44	0.976	<0.01	92.5	97.5

**Figure 7:**
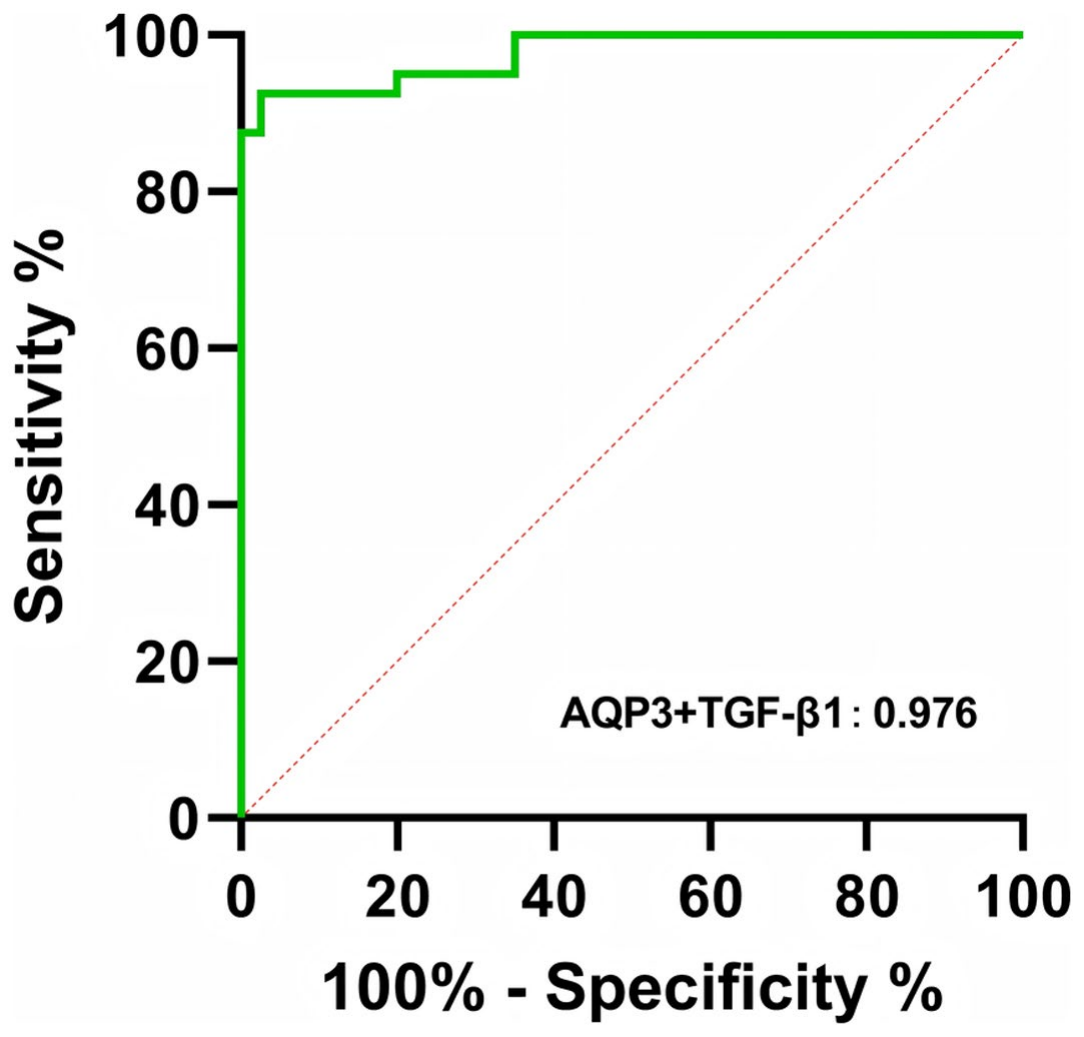
ROC curve of combined serum AQP3 and TGF-β1 for the diagnosis of MA.

The final model combining AQP3 and TGF-β1 showed an AUC of 0.9763, indicating enhanced diagnostic accuracy. The optimal diagnostic cutoff value for this combined model was 0.44 ng/L (Figure 7; Table 2). The logistic regression model is as follows:


$$\mathrm{logit}\left(P\right)=-9.497+1.825\times \left(\mathrm{AQP3 value}\right)+3.469\times \left(TGF-\beta \mathrm{1 value}\right)$$

The *p* value represents the predicted probability of MA. This model can be used to assess the risk of MA in patients and provide valuable information for clinical decision-making.

## 4 Discussion

The causes of MA are complex and multifactorial, encompassing environmental influences and individual physiological conditions (32). These factors are interrelated, with each potentially affecting the others. During the first trimester of pregnancy, differentiated extravillous trophoblasts (EVTs) invade maternal blood vessels, facilitating placental anchorage and remodeling spiral arteries to enhance blood flow in the chorionic space, thereby supporting successful embryo implantation (11, 12). Effective EVT invasion into the endometrium is crucial for maintaining pregnancy and shares mechanistic similarities with tumor cell invasion (13).

Throughout the processes of placental implantation and formation, the endometrial matrix and trophoblast basement membrane undergo selective degradation (33). The depth of trophoblast invasion is not only critical for the implantation of early embryos but also influences the decidualization of the endometrium (34). Consequently, the ability of trophoblasts to invade the endometrium effectively is vital for the normal progression of human pregnancy. Abnormal trophoblast invasion and impaired angiogenesis at the maternal-fetal interface can lead to implantation failure and subsequent embryo development issues, resulting in pregnancy loss (35).

Endometrial decidualization is a critical process for the establishment and maintenance of pregnancy. It involves several key changes, including increased secretion from endometrial glandular cells, the activation of local immune cells, remodeling of spiral arteries, and alterations in extracellular matrix components (36). This process serves two primary functions: first, it provides essential nutrients for embryonic development prior to the formation of the placenta; second, it prevents the maternal immune system from rejecting the embryo, which is recognized as a semi-allograft (37). Disruptions in the decidualization process can result in the implantation of poor-quality embryos within the uterine cavity, leading to implantation failure, embryonic dysplasia, and early abortion (38). Thus, proper decidualization is crucial for ensuring a successful pregnancy.

AQP3 plays a vital role in mammalian pregnancy, contributing to gamete formation, embryo implantation, placental development, and the maintenance of normal pregnancy (39). Inhibiting AQP3 function significantly impairs the migration of extravillous trophoblasts (EVTs), underscoring its necessity for this process (24). During early pregnancy, AQP3 is crucial for trophoblast invasion and embryo implantation. Notably, increased AQP3 expression may induce programmed cell death, while decreased AQP3 levels could represent an adaptive response by the placenta to mitigate trophoblast apoptosis (23, 40). In our study, we observed a marked increase in AQP3 expression in the decidua tissue of patients with MA compared to those undergoing IA, as evidenced by both protein and mRNA analyses. We hypothesize that the overexpression of AQP3 may elevate EVT apoptosis, leading to impaired embryo implantation and development, ultimately contributing to the occurrence of MA.

TGF-β1 is a multifunctional cytokine primarily secreted by CD4+ T cells, exerting effects on surrounding tissues through both autocrine and paracrine mechanisms (25). It plays a critical role in cell proliferation and differentiation, extracellular matrix production, and the regulation of trophoblast proliferation, differentiation, and immune function (26, 31). In our study, we observed that TGF-β1 expression in the decidua tissue of patients with MA was significantly higher than that in patients undergoing IA. This increased expression may lead to impaired trophoblast invasion, hindering normal embryo survival. Several potential mechanisms may contribute to this phenomenon: (1) regulation of trophoblast invasion: TGF-β1 may modulate trophoblast invasion into the endometrium; (2) induction of TIMP-1 production: TGF-β1 can induce the production of tissue inhibitor of metalloproteinase-1 (TIMP-1), which inhibits trophoblast invasion (41); (3) suppression of MMP-9 secretion: TGF-β1 may reduce the secretion of matrix metalloproteinase-9 (MMP-9), consequently diminishing the invasive capacity of villous trophoblasts (41). We hypothesize that the elevated levels of TGF-β1 may exert a stronger inhibitory effect on MMP-9, resulting in shallow trophoblast invasion, subsequent necrosis and detachment of decidual tissue, and ultimately, failure of embryo implantation. Thus, TGF-β1 appears to play a crucial role in the processes surrounding embryo implantation.

Based on the results of this study, we found that the expression of AQP3 and TGF-β1 in the decidual tissue of MA patients was significantly higher than that in IA patients. This finding suggests that AQP3 and TGF-β1 may play an important role in the pathogenesis of MA. Immunohistochemistry (IHC) and Western blot (WB) analyses further confirmed these results, with good consistency between the two methods. This indicates that the elevated protein expression of AQP3 and TGF-β1 in MA patients may reflect their pathophysiological roles in the disease.

To further explore the mechanisms of AQP3 and TGF-β1, we assessed the mRNA expression levels of AQP3 and TGF-β1 in decidual tissue using RT-qPCR. The results supported the conclusions from IHC and WB analyses. The mRNA levels of AQP3 and TGF-β1 in the MA group were significantly higher than those in the IA group, suggesting that AQP3 and TGF-β1 are not only elevated at the protein level but also upregulated at the gene transcription level, potentially contributing to the onset and progression of MA.

Given the high expression of AQP3 and TGF-β1 in decidual tissue, we further investigated the expression of these biomarkers in serum. Using ELISA, we measured the serum levels of AQP3 and TGF-β1. The results revealed that serum levels of AQP3 and TGF-β1 were significantly higher in the MA group, with increases of 2.3-fold and 3.3-fold, respectively, compared to the IA group. This finding suggests that serum AQP3 and TGF-β1 may serve as potential biomarkers for the diagnosis of MA.

We then evaluated the diagnostic accuracy of AQP3 and TGF-β1 as biomarkers for MA using ROC curve analysis. When analyzed individually, both AQP3 (AUC = 0.8869) and TGF-β1 (AUC = 0.9494) demonstrated high diagnostic accuracy, indicating their promising clinical application in predicting MA risk. However, ROC analysis of AQP3 revealed multiple thresholds with the same maximal Youden index, making it challenging to select a single optimal diagnostic threshold. To improve prediction accuracy, we combined AQP3 and TGF-β1 into a predictive model, which resulted in an AUC of 0.9763, demonstrating a significant improvement in diagnostic accuracy.

Using logistic regression analysis, we developed a predictive model based on the serum levels of AQP3 and TGF-β1, with an optimal diagnostic threshold of 0.44 ng/L. This model not only improved the accuracy of MA prediction but also provides clinicians with a practical tool for assessing the risk of MA. The combined prediction of AQP3 and TGF-β1 may offer new biomarkers and diagnostic criteria for the early diagnosis and clinical management of MA.

In conclusion, this study provides strong evidence supporting AQP3 and TGF-β1 as potential biomarkers for MA. The established combined diagnostic model has high clinical application value. Future research should further validate the applicability of this model in different populations and explore the specific roles of AQP3 and TGF-β1 in the pathogenesis of MA.

## 5 Limitations of the study

Although this study provides preliminary evidence supporting AQP3 and TGF-β1 as potential biomarkers for MA, several limitations need to be addressed in future research.

Firstly, the sample size in this study was relatively small, which may affect the generalizability and statistical robustness of the results. While we obtained consistent conclusions across multiple experimental methods (immunohistochemistry, RT-qPCR, ELISA, etc.), the small sample size may introduce random variation or bias in the findings. Therefore, future studies should expand the sample size, particularly by validating the results in diverse populations, to enhance the representativeness and credibility of the findings.

Secondly, the data in this study were collected from a single hospital and a single region, which could be influenced by regional or population-specific differences. Patients from different regions, ethnicities, or age groups may exhibit variations in the expression levels of AQP3 and TGF-β1, potentially affecting the external validity of the results. To address this, future research should involve samples from multiple regions and hospitals, helping to eliminate potential regional and demographic confounding factors and improving the broader applicability of the results.

Additionally, although this study has validated the diagnostic potential of AQP3 and TGF-β1 through ROC curve analysis, it is limited to serum level measurements. The expression of AQP3 and TGF-β1 is regulated by various factors, such as immune responses and inflammatory processes. Therefore, relying solely on these two biomarkers may not fully capture the complex pathological mechanisms of MA. Future studies could consider integrating other clinical indicators or molecular biomarkers to construct a more comprehensive diagnostic model, enhancing both the accuracy and sensitivity of diagnosis.

Finally, while the logistic regression model used in this study showed a high AUC, its clinical applicability still needs further validation. Larger-scale prospective studies are necessary to assess the long-term stability and predictive power of the model to ensure its effectiveness in real-world diagnostic settings. Additionally, practical considerations, such as detection methods, cost, and the availability of detection equipment, should also be taken into account.

In conclusion, while this study provides preliminary evidence for the use of AQP3 and TGF-β1 as biomarkers for MA diagnosis, further large-scale, multi-center studies are needed to validate their generalizability and clinical value. Incorporating other relevant factors will also be essential to further improve diagnostic accuracy.

## Data Availability

The original contributions presented in the study are included in the article/supplementary material, further inquiries can be directed to the corresponding author.

## References

[1] Tian Q Xia S Wu Y Zhang J Wang L Zhu W. Comprehensive analysis of the differential expression profile of microRNAs in missed abortion. Kaohsiung J Med Sci. (2020) 36:114–21. doi: 10.1002/kjm2.12144, PMID: 31688986 PMC11896336

[2] Xiao Q Zeng F Tang G Lei C Zou X Liu X. Expression of galectin3 and apoptosis in placental villi from patients with missed abortion during early pregnancy. Exp Ther Med. (2019) 17:2623–31. doi: 10.3892/etm.2019.7227, PMID: 30906454 PMC6425259

[3] Luan X Yan Y Zheng Q Wang M Chen W Yu J. Excessive reactive oxygen species induce apoptosis via the APPL1-Nrf2/HO-1 antioxidant signalling pathway in trophoblasts with missed abortion. Life Sci. (2020) 254:117781. doi: 10.1016/j.lfs.2020.117781, PMID: 32407842

[4] Li Y Liu X Sun Y Liu Y Wan L Zhang L. The expression of PDCD4 in patients with missed abortion and its clinical significance. Reprod Sci. (2017) 24:1512–9. doi: 10.1177/1933719117692044, PMID: 29017439

[5] Fu M Mu S Wen C Jiang S Li L Meng Y. Wholeexome sequencing analysis of products of conception identifies novel mutations associated with missed abortion. Mol Med Rep. (2018) 18:2027–32. doi: 10.3892/mmr.2018.9201, PMID: 29956774 PMC6072200

[6] Zhang L Liu W Hou K Lin J Zhou C Tong X. Air pollution-induced missed abortion risk for pregnancies. Nat Sustain. (2019) 2:1011–7. doi: 10.1038/s41893-019-0387-y

[7] Segawa T Kuroda T Kato K Kuroda M Omi K Miyauchi O. Cytogenetic analysis of the retained products of conception after missed abortion following blastocyst transfer: a retrospective, large-scale, single-Centre study. Reprod Biomed Online. (2017) 34:203–10. doi: 10.1016/j.rbmo.2016.11.005, PMID: 27913136

[8] Philipp T Philipp K Reiner A Beer F Kalousek DK. Embryoscopic and cytogenetic analysis of 233 missed abortions: factors involved in the pathogenesis of developmental defects of early failed pregnancies. Hum Reprod. (2003) 18:1724–32. doi: 10.1093/humrep/deg309, PMID: 12871891

[9] Choi H Choi BC Lee S Kim JW Cha KY Baek K. Expression of angiogenesis- and apoptosis-related genes in chorionic villi derived from recurrent pregnancy loss patients. Mol Reprod Dev. (2003) 66:24–31. doi: 10.1002/mrd.10331, PMID: 12874795

[10] Ozbilgin K Karaca F Turan A Köse C Vatansever S Ozcakir T. The higher heparin-binding epidermal growth factor (HB-EGF) in missed abortion. Taiwan J Obstet Gynecol. (2015) 54:13–8. doi: 10.1016/j.tjog.2013.08.011, PMID: 25675913

[11] Menkhorst E Winship A Van Sinderen M Dimitriadis E. Human extravillous trophoblast invasion: intrinsic and extrinsic regulation. Reprod Fertil Dev. (2016) 28:406–15. doi: 10.1071/RD14208, PMID: 25163485

[12] Moser G Weiss G Sundl M Gauster M Siwetz M Lang-Olip I. Extravillous trophoblasts invade more than uterine arteries: evidence for the invasion of uterine veins. Histochem Cell Biol. (2017) 147:353–66. doi: 10.1007/s00418-016-1509-5, PMID: 27774579 PMC5344955

[13] Dakouane-Giudicelli M Brouillet S Traboulsi W Torre A Vallat G Si Nacer S. Inhibition of human placental endothelial cell proliferation and angiogenesis by netrin-4. Placenta. (2015) 36:1260–5. doi: 10.1016/j.placenta.2015.09.007, PMID: 26390805

[14] He N van Iperen L de Jong D Szuhai K Helmerhorst FM van der Westerlaken LAJ. Human extravillous trophoblasts penetrate decidual veins and lymphatics before remodeling spiral arteries during early pregnancy. PLoS One. (2017) 12:e0169849. doi: 10.1371/journal.pone.0169849, PMID: 28081266 PMC5230788

[15] Windsperger K Dekan S Pils S Golletz C Kunihs V Fiala C. Extravillous trophoblast invasion of venous as well as lymphatic vessels is altered in idiopathic, recurrent, spontaneous abortions. HumReprod. (2017) 32:1208–17. doi: 10.1093/humrep/dex058, PMID: 28369440

[16] Burke SD Karumanchi SA. Spiral artery remodeling in preeclampsia revisited. Hypertension. (2013) 62:1013–4. doi: 10.1161/HYPERTENSIONAHA.113.02049, PMID: 24144648

[17] Brosens I Puttemans P Benagiano G. Placental bed research: I. The placental bed: from spiral arteries remodeling to the great obstetrical syndromes. Am J Obstet Gynecol. (2019) 221:437–56. doi: 10.1016/j.ajog.2019.05.044, PMID: 31163132

[18] Kasa P Farran B Prasad GLV Nagaraju GP. Aquaporins in female specific cancers. Gene. (2019) 700:60–4. doi: 10.1016/j.gene.2019.03.032, PMID: 30898710

[19] Marlar S Jensen HH Login FH Nejsum LN. Aquaporin-3 in Cancer. Int J Mol Sci. (2017) 18:2106. doi: 10.3390/ijms18102106, PMID: 28991174 PMC5666788

[20] Martínez N Damiano AE. Aquaporins in fetal development In: Yang B, editor. Aquaporins. Dordrecht: Springer (2017). 199–212. 10.1007/978-94-024-1057-0_13 28258575

[21] Damiano AE. Chapter fifteen - Aquaporins during pregnancy In: Litwack G, editor. Vitamins and hormones: Academic Press (2020). 327–55. Available at: https://www.sciencedirect.com/science/article/abs/pii/S008367291930069X?via%3Dihub 10.1016/bs.vh.2019.08.009 32061348

[22] Zhang C Li Y Wang J Liu C Chen Y. Association between levels of aquaporin 3 in the placenta and adiponectin in the umbilical cord blood with gestational diabetes mellitus and pregnancy outcome. Mol Med Rep. (2020) 22:1498–506. doi: 10.3892/mmr.2020.11225, PMID: 32627013 PMC7339817

[23] Szpilbarg N Damiano AE. Expression of aquaporin-3 (AQP3) in placentas from pregnancies complicated by preeclampsia. Placenta. (2017) 59:57–60. doi: 10.1016/j.placenta.2017.09.010, PMID: 29108637

[24] Alejandra R Natalia S Alicia E D. The blocking of aquaporin-3 (AQP3) impairs extravillous trophoblast cell migration. Biochem Bioph Res Commun. (2018) 499:227–32. doi: 10.1016/j.bbrc.2018.03.133, PMID: 29567477

[25] Budi EH Xu J Derynck R. Regulation of TGF-β receptors In: Feng X-H Xu P Lin X, editors. TGF-β signaling: Methods and protocols. New York, NY: Springer New York (2016). 1–33.

[26] David CJ Massagué J. Contextual determinants of TGFβ action in development, immunity and cancer. Nat Rev Mol Cell Biol. (2018) 19:419–35. doi: 10.1038/s41580-018-0007-0, PMID: 29643418 PMC7457231

[27] Derynck R Jarrett JA Chen EY Eaton DH Bell JR Assoian RK. Human transforming growth factor-β complementary DNA sequence and expression in normal and transformed cells. Nature. (1985) 316:701–5. doi: 10.1038/316701a0, PMID: 3861940

[28] Brooks SA Martin E Smeester L Grace MR Boggess K Fry RC. miRNAs as common regulators of the transforming growth factor (TGF)-β pathway in the preeclamptic placenta and cadmium-treated trophoblasts: links between the environment, the epigenome and preeclampsia. Food Chem Toxicol. (2016) 98:50–7. doi: 10.1016/j.fct.2016.06.023, PMID: 27375191 PMC5156314

[29] Shooner C Caron P-L Fréchette-Frigon G Leblanc V Déry M-C Asselin E. TGF-beta expression during rat pregnancy and activity on decidual cell survival. Reprod Biol Endocrinol. (2005) 3:20. doi: 10.1186/1477-7827-3-20, PMID: 15927076 PMC1166574

[30] Farhadi E Mahmoudi M Rahmani F Yousefi B Sarafnejad A Kavosi H. Attenuation of aquaporin-3 and epidermal growth factor receptor expression and activation in systemic sclerosis dermal fibroblasts. J Cell Physiol. (2019) 234:12876–83. doi: 10.1002/jcp.27952, PMID: 30536805 PMC13483328

[31] Luo J Liu X Liu J Jiang M Luo M Zhao J. Activation of TGF-β1 by AQP3-mediated H_2_O_2_ transport into fibroblasts of a bleomycin-induced mouse model of scleroderma. J Invest Dermatol. (2016) 136:2372–9. doi: 10.1016/j.jid.2016.07.014, PMID: 27456753

[32] Hu Y Huo ZH Liu CM Liu SG Zhang N Yin KL. Functional study of one nucleotide mutation in pri-MiR-125a coding region which related to recurrent pregnancy loss. PLoS One. (2014) 9:e114781. doi: 10.1371/journal.pone.0114781, PMID: 25479352 PMC4257728

[33] Cartwright J Fraser R Leslie K Wallace A James J. Remodelling at the maternal-fetal interface: relevance to human pregnancy disorders. Reproduction. (2010) 140:803–13. doi: 10.1530/REP-10-0294, PMID: 20837731

[34] Ji L Brkić J Liu M Fu G Peng C Wang Y-L. Placental trophoblast cell differentiation: physiological regulation and pathological relevance to preeclampsia. Mol Asp Med. (2013) 34:981–1023. doi: 10.1016/j.mam.2012.12.008, PMID: 23276825

[35] George EM Granger JP. Recent insights into the pathophysiology of preeclampsia. Exp Rev Obstetr Gynecol. (2010) 5:557–66. doi: 10.1586/eog.10.45, PMID: 21170149 PMC3001629

[36] Evans J Salamonsen LA. Decidualized human endometrial stromal cells are sensors of hormone withdrawal in the menstrual inflammatory cascade. Biol Reprod. (2014) 90:1–12. doi: 10.1095/biolreprod.113.108175, PMID: 24227758

[37] Wagner G Kin K Muglia L Pavli M. Evolution of mammalian pregnancy and the origin of the decidual stromal cell. Int J Dev Biol. (2014) 58:117–26. doi: 10.1387/ijdb.130335gw, PMID: 25023677

[38] Salker M Teklenburg G Molokhia M Lavery S Trew G Aojanepong T. Natural selection of human embryos: impaired decidualization of endometrium disables embryo-maternal interactions and causes recurrent pregnancy loss. PLoS One. (2010) 5:e10287. doi: 10.1371/journal.pone.0010287, PMID: 20422017 PMC2858209

[39] Cui D Sui L Han X Zhang M Guo Z Chen W. Aquaporin-3 mediates ovarian steroid hormone-induced motility of endometrial epithelial cells. Hum Reprod. (2018) 33:2060–73. doi: 10.1093/humrep/dey290, PMID: 30285121 PMC6195804

[40] Szpilbarg N Castro-Parodi M Reppetti J Repetto M Maskin B Martinez N. Placental programmed cell death: insights into the role of aquaporins. Mol Hum Reprod. (2015) 22:46–56. doi: 10.1093/molehr/gav063, PMID: 26568619

[41] Singh MK Bhattacharya D Chaudhuri S Acharya S Kumar P Santra P. T11TS inhibits glioma angiogenesis by modulation of MMPs, TIMPs, with related integrin αv and TGF-β1 expressions. Tumour Biol. (2014) 35:2231–46. doi: 10.1007/s13277-013-1296-8, PMID: 24242015

